# Netrin-1-Induced Stem Cell Bioactivity Contributes to the Regeneration of Injured Tissues via the Lipid Raft-Dependent Integrin α6β4 Signaling Pathway

**DOI:** 10.1038/srep37526

**Published:** 2016-11-24

**Authors:** Soo Sang Lee, Sei-Jung Lee, Sang Hun Lee, Jung Min Ryu, Hyeon Su Lim, Jun Sung Kim, Eun Ju Song, Young Hyun Jung, Hyun Jik Lee, Chung Hun Kim, Ho Jae Han

**Affiliations:** 1 Department of plastic and reconstructive surgery, Bundang CHA Medical Center, Yatap-dong, Bundang-gu, Seongnam-si, Gyeonggi-do, Korea; 2 SKY plastic surgery clinic, 4F, 826-23, Yeoksam-dong, Gangnam-gu, Seoul, Korea; 3 Department of Veterinary Physiology, College of Veterinary Medicine, Research Institute for Veterinary Science and BK21 PLUS Program for Creative Veterinary Science Research Center, Seoul National University, Seoul 08826, Korea; 4 Medical Science Research Institute, Soonchunhyang University Seoul Hospital, Seoul, Korea; 5 Department of Veterinary Physiology, College of Veterinary Medicine, Chonnam National University, Gwangju, Republic of Korea

## Abstract

Netrin-1 (Ntn-1) is a multifunctional neuronal signaling molecule; however, its physiological significance, which improves the tissue-regeneration capacity of stem cells, has not been characterized. In the present study, we investigate the mechanism by which Ntn-1 promotes the proliferation of hUCB-MSCs with regard to the regeneration of injured tissues. We found that Ntn-1 induces the proliferation of hUCB-MSCs mainly via Inα6β4 coupled with c-Src. Ntn-1 induced the recruitment of NADPH oxidases and Rac1 into membrane lipid rafts to facilitate ROS production. The Inα6β4 signaling of Ntn-1 through ROS production is uniquely mediated by the activation of SP1 for cell cycle progression and the transcriptional occupancy of SP1 on the VEGF promoter. Moreover, Ntn-1 has the ability to induce the F-actin reorganization of hUCB-MSCs via the Inα6β4 signaling pathway. In an *in vivo* model, transplantation of hUCB-MSCs pre-treated with Ntn-1 enhanced the skin wound healing process, where relatively more angiogenesis was detected. The potential effect of Ntn-1 on angiogenesis is further verified by the mouse hindlimb ischemia model, where the pre-activation of hUCB-MSCs with Ntn-1 significantly improved vascular regeneration. These results demonstrate that Ntn-1 plays an important role in the tissue regeneration process of hUCB-MSC via the lipid raft-mediated Inα6β4 signaling pathway.

Stem cell therapy has been emphasized as a promising adjunct for improving the regeneration of injured tissues in translational medicine^1^,^2^,^3^. Many studies have recently announced that an improved proliferation capacity together with the migration and differentiation of stem cells at damaged tissues is necessary for tissue regeneration^1^,^2^,^3^. In fact, adult stem cells reside in specific microenvironments known as niches, which play important roles in the modulation of stem cell behaviors^4^, and many therapeutic approaches have focused on the manipulation of stem cell niches using biochemical cocktails to enhance the therapeutic potential of stem cells^5^,^6^. However, these methods are considered impractical for clinical use due to unexpected potential side effects. In this respect, the identification of a regulating factor to activate stem cells could provide a new therapeutic strategy for the success of stem cell therapy when used for tissue regeneration. Therefore, the development of safe, effective, and practical means by which to improve stem cell proliferation is a priority in the research on effective tissue regeneration therapies.

Netrin-1 (Ntn-1), a diffusible neural guidance protein, is a bifunctional signaling molecule related to cell proliferation, cell migration, and cell-extracellular matrix adhesion during the neuronal development process^7^,^8^. Although the Ntn-1 signaling pathway in non-neuronal tissue has received little attention, many netrin receptors have been detected, not only in the nervous system^7^,^9^, but also in a number of other tissues^8^,^10^, where their functions remain largely unknown. We recently described the expression of Ntn-1 receptors in mesenchymal stem cells (MSCs), where Ntn-1 plays a major role in the control of cell motility and survival by interacting with the deleted in colorectal cancer (DCC) and integrin (In)α6β4 receptors^11^,^12^. Recent reports have also revealed that transplantation of bone-marrow MSCs infected by a recombinant adenovirus expressing Ntn-1 improved functional recovery from peripheral nerve injuries^13^. Despite the fact that Ntn-1 has attracted much attention regarding its potential in cell proliferation and tissue regeneration capacities^10^,^13^,^14^,^15^, the underlying cellular mechanisms of Ntn-1, which improves the bioactivity of stem cells during the tissue regeneration process, remain largely unknown.

Therapeutic applications of human umbilical-cord-blood-derived (hUCB) MSCs have been recognized as promising in the treatment of clinical diseases [18]. hUCB-MSCs have multi-lineage differentiation potential and low immunogenicity, and they can be isolated from the umbilical cord vein of the placenta after detachment from a newborn, remaining free from any ethical controversies^16^,^17^,^18^. Specifically, hUCB-MSCs have been shown to improve wound healing in several studies^19^,^20^. Due to the functional relevance of Ntn-1 in regulating placental angiogenesis^21^, hUCB-MSCs are likely to be important to those attempting to define the role of Ntn-1 in the tissue regeneration of stem cells. In this study, we therefore investigate the mechanism of the Ntn-1 signaling pathway in promoting the bioactivity of hUCB-MSCs with regard to the regeneration of injured tissues.

## Results

### Regulatory effect of Ntn-1 on stem cell proliferation

**Figure 1:**
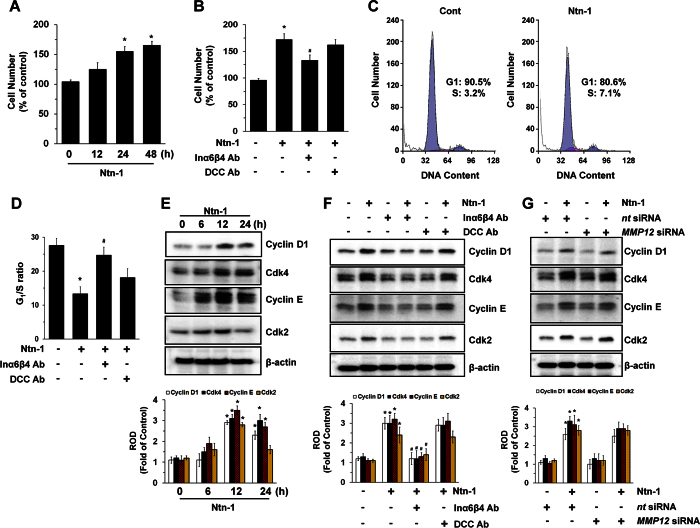
Regulatory effect of Ntn-1on stem cell proliferation. (**A**) hUCB-MSCs were incubated with 50 ng/mL of Ntn-1 for 48 h, and the number of cells was counted. Data represent the mean ± S.E. *n* = 5. *P < 0.05 vs. 0 h (**B**) Cells were pre-treated with DCC-function-blocking antibody (2.5 μg/mL) or a combination of Inα6- and Inβ4-function-blocking antibodies (2.5 μg/mL) for 30 min prior to Ntn-1 exposure for 24 h. Cell counting was performed. Data represent the mean ± S.E. *n* = 4. *P < 0.05 vs. vehicle. ^*#*^*P* < 0.05 vs. Ntn-1 alone. (**C**) Cells were treated with Ntn-1 for 24 h. Gates were manually configured to determine the percentage of cells in S phase based on DNA content by using PI staining and flow cytometry. Data represent the mean ± S.E. *n* = 4. (**D**) G_1_/S ratios measured by flow cytometry. Data represent the mean ± S.E. *n* = 4. *P < 0.01 vs. vehicle. ^*#*^*P* < 0.05 vs. Ntn-1 alone. (**E**) The cells were incubated in the presence of Ntn-1 for 24 h and then harvested. Total protein was extracted and blotted with Cyclin D1, CDK4, Cyclin E, and CDK2 antibodies. Data represent the mean ± S.E. *n* = 4. *P < 0.05 vs. 0 h. (**F**) The level of cell cycle proteins in cells pre-treated with DCC-function-blocking antibody (2.5 μg/mL) or a combination of Inα6- and Inβ4-function-blocking antibodies (2.5 μg/mL) for 30 min prior to Ntn-1 exposure for 24 h is shown. Data represent the mean ± S.E. *n* = 4. *P < 0.01 vs. vehicle. ^*#*^*P* < 0.05 vs. Ntn-1 alone. (**G**) The effect of Ntn-1 on the level of cell cycle proteins in cells transfected with *MMP12*siRNA is shown. Cells were transfected for 24 h with specific siRNA for *MMP12* prior to Ntn-1 exposure for 24 h. *Non-targeting (nt)* control siRNA was used as a negative control. Data represent the mean ± S.E. *n* = 4. *P < 0.01 vs. *nt* siRNA. (**E**–**G**) ROD is the abbreviation for relative optical density.

To determine the functional role of netrin-1 (Ntn-1), hUCB-MSCs were exposed to Ntn-1 for 48 h. Ntn-1 significantly increased the number of hUCB-MSC from 24 h to 48 h at 50 ng/mL in a time-dependent manner (Fig. 1A). The activity of Ntn-1 to promote cell proliferation was significantly attenuated by a pre-treatment with a combination of Inα6- and Inβ4-function-blocking antibodies (2.5 μg/mL), but not by a DCC-function-blocking antibody (2.5 μg/mL), suggesting that Ntn-1 has receptor specificity in promoting cell proliferation (Fig. 1B). We previously confirmed that Inα6β4- and DCC-function-blocking antibodies have significant capacities to block the Inα6β4 and DCC functions when regulating stem cell motility^11^ and apoptosis^12^. A flow cytometric analysis revealed that Ntn-1 induced a G_1_-to-S-phase transition (Fig. 1C), resulting in a significant decrease in the G_1_/S ratio for 24 h in an Inα6β4-dependent manner (Fig. 1D). Cell cycle progression is regulated by protein complexes composed of cyclins and cyclin-dependent kinases (CDKs)^22^. Ntn-1 increased the levels of Cyclin D1, Cdk4, Cyclin E, and Cdk2 (Fig. 1E). The blocking of Inα6β4 activity, but not DCC, decreased the amounts of cyclins and CDKs (Fig. 1F). Knockdown of MMP-12, which is known to enhance cell migration in response to Ntn-1^11^, was not found to have an effect on the levels of Cyclin D1, Cdk4, Cyclin E, or Cdk2 (Fig. 1G), indicating that Ntn-1 uniquely mediates cell-cycle transition from the G_1_ to the S phase via Inα6β4-dependent pathways in promoting hUCB-MSC proliferation.

### Regulatory effect of Ntn-1 on the activation of c-Src and Rac1

**Figure 2:**
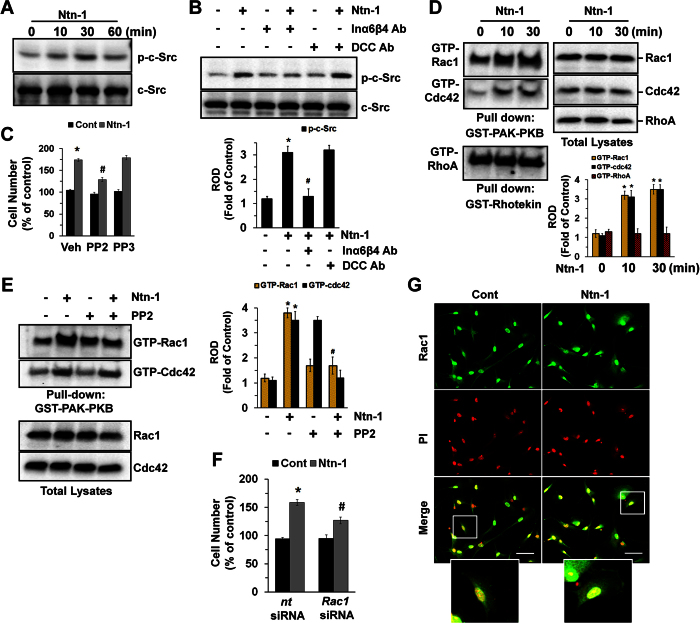
Regulatory effect of Ntn-1 on activation of c-Src and Rac1. (**A**) Phosphorylation of c-Src in cells treated with Ntn-1 for 60 min is shown. (**B**) Phosphorylation of c-Src in cells pre-treated with DCC-function-blocking antibody or a combination of Inα6- and Inβ4-function-blocking antibodies for 30 min prior to Ntn-1 exposure for 30 min is shown. Data represent the mean ± S.E. *n* = 4. *P < 0.01 vs. vehicle. ^*#*^*P* < 0.01 vs. Ntn-1 alone. (**C**) Cells were pre-treated with PP2 (10 μM) or PP3 (10 μM) for 30 min prior to Ntn-1 exposure for 24 h. Cell counting with a hemocytometer was performed. Data represent the mean ± S.E. *n* = 4. *P < 0.01 vs. vehicle alone. ^*#*^*P* < 0.05 vs. Ntn-1 + vehicle alone. (**D**) Cells were treated with Ntn-1 for 30 min, and the lysates (400 μg) were incubated with agarose beads containing GST-PAK-PBD or GST-Rhotekin-RBD. The bound activated GTP-Rac1, GTP-Cdc42, and GTP-RhoA were resolved by SDS-PAGE, transferred, and blotted using an anti-Rac1, anti-Cdc42, and anti-RhoA antibodies to determine the extent of the activation of Rac1, Cdc42, and RhoA. Total Rac1, Cdc42, and RhoA levels were determined using lysates (right panel). Data represent the mean ± S.E. *n* = 4. *P < 0.05 vs. vehicle. (**E**) The activation of Rac1 and Cdc42 in cells pre-treated with PP2 (10 μM) for 30 min prior to Ntn-1 exposure for 30 min is shown. Data represent the mean ± S.E. *n* = 4. *P < 0.01 vs. vehicle. ^*#*^*P* < 0.05 vs. Ntn-1 alone. (**F**) Cells were transfected with *Rac1*siRNA prior to Ntn-1 exposure for 24 h. Cell counting with a hemocytometer was performed. Data represent the mean ± S.E. *n* = 4. *P < 0.01 vs. *nt* siRNA alone. ^*#*^*P* < 0.05 vs. *nt* siRNA + Ntn-1 alone (**G**) Rac1 expression (green) was determined by confocal microscopy using immunofluorescence staining. Propidium iodide (PI) was used for nuclear counterstaining (red). Scale bars, 100 μm (magnification, x400). *n* = 3. (**B**–**E**) ROD is the abbreviation for relative optical density.

Src tyrosine kinases transmit integrin-dependent signals that are necessary for cell proliferation and movement^23^. Ntn-1 (50 ng/mL) significantly induced the phosphorylation of c-Src at the Tyr 419 site in 30 min (Fig. 2A). c-Src activation was markedly blocked by a pre-treatment with a combination of Inα6- and Inβ4-function-blocking antibodies (2.5 μg/mL) (Fig. 2B). Interestingly, a pre-treatment with the Src family kinase inhibitor PP2 inhibited the phosphorylation of c-Src (Supplementary Fig. 1A) and cell proliferation as induced by Ntn-1 (Fig. 2C). However, the enhanced level of c-Src activation (Supplementary Fig. 1B) and cell proliferation (Fig. 2C) were not affected by treatment with PP3, an inactive analogue of PP2. Rho GTPases are the major effector of c-Src^24^,^25^. Affinity precipitation for small Rho GTPases has revealed that Ntn-1 stimulates the activity of Rac1 and Cdc42, but not RhoA (Fig. 2D). PP2, however, significantly blocked the Ntn-1-induced activation of Rac1, but not Cdc42, suggesting that Rac1 activation is regulated by c-Src family kinases, including c-Src (Fig. 2E). Moreover, PP3 did not have an effect on Rac1/Cdc42 activation (Supplementary Fig. 1C), and the silencing of *Rac1* with *Rac1*siRNA inhibited Ntn-1-induced cell proliferation (Fig. 2F). The translocation of Rac1 to the leading edges of lamellipodia was also observed after cells were treated with Ntn-1 for 30 min (Fig. 2G). These findings indicate that c-Src phosphorylation is involved in the Rac1 activation and cell proliferation processes.

### Cell proliferation is mediated by lipid raft-mediated ROS production

**Figure 3:**
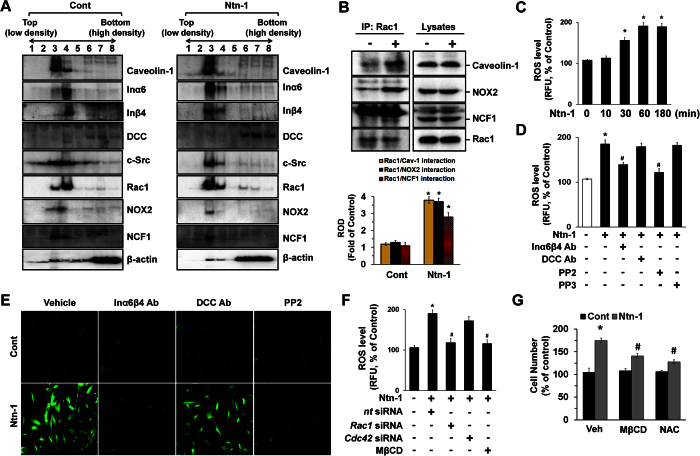
Cell proliferation is mediated by lipid raft-mediated ROS production. (**A**) Caveolin-enriched membrane fractions were prepared by discontinuous sucrose density gradient fractionation, and the location of Caveolin-1, Inα6, Inβ4, DCC, c-Src, Rac1, NOX2, and NCF1 was determined by Western blot analysis. *n* = 3. (**B**) Rac1 co-immunoprecipitated with caveolin-1, NOX2, and NCF1 is shown (left side). The level of caveolin-1, NOX2, NCF1 and Rac1 in total cell lysates is shown in the right side. (**C**) Time responses of 50 ng/mL of Ntn-1 for 180 min in ROS production are shown. *n* = 5. (**D**) The level of ROS production in cells pre-treated with DCC-function-blocking antibody, a combination of Inα6- and Inβ4-function-blocking antibodies, PP2 (10 μM), or PP3 (10 μM) for 30 min prior to Ntn-1 exposure for 60 min is shown. Data represent the mean ± S.E. *n* = 4. *P < 0.01 vs. vehicle. ^*#*^*P* < 0.05 vs. Ntn-1 alone. (**E**) ROS production (green) was visualized by confocal microscopy. Scale bars, 100 μm. *n* = 4. (**F**) The level of ROS production in cells transfected with siRNA for *Rac1* and *Cdc42* or pre-treated with MβCD (0.1 mM) was shown. Data represent the means ± S.E. *P < 0.01 vs. vehicle. ^*#*^*P* < 0.01 vs. *nt* siRNA + Ntn-1. (**G**) Cells were pre-treated with MβCD (0.1 mM) and NAC (10 μM) for 30 min prior to Ntn-1 exposure for 24 h. Cell counting with a hemocytometer was performed. Data represent the mean ± S.E. *n* = 4. *P < 0.05 vs. vehicle alone. ^*#*^*P* < 0.05 vs. vehicle + Ntn-1 alone. (**B**–**D**,**F**) ROD and RFU are the abbreviations for relative optical density and relative fluorescence unit, respectively.

Membrane lipid rafts are key platforms regulating the signaling pathways of integrin and Ntn-1 to trigger anchorage-dependent cell growth^26^ and to mediate growth cone guidance^27^, respectively. Figure 3A shows that Inα6 and Inβ4 were detected in fractions 3 and 4, enriched in Caveolin-1, which is a major marker of lipid rafts. However, the membrane location of DCC was in the non-lipid raft part. Importantly, Ntn-1 markedly induced the recruitment of Inα6 and Inβ4 into fraction 3, suggesting that a subcellular location of the Ntn-1 receptors is critical for the regulation of hUCB-MSC proliferation. Moreover, c-Src, Rac1, and the subunits of the NADPH oxidase (NOX) enzymes NOX2 and NCF1 were highly enriched in fractions 3 and 4. However, a Ntn-1 treatment resulted in the translocations of c-Src, Rac1, NOX2, and NCF1 into fraction 3. Because the above approaches are qualitative at best, we also attempted to quantify the results using the co-immunoprecipitation of Rac1 with proteins related to the lipid rafts in the presence of Ntn-1 (Fig. 3B). It was noted that Rac1 co-immunoprecipitated with Caveolin-1, NOX2, and NCF1, and importantly, that these interactions were enhanced by the Ntn-1 treatment. This indicates that the lipid raft-mediated clustering of Rac1, NOX2, and NCF1may be required to trigger the Ntn-1 signaling pathway. To confirm the functional importance of these interactions, we further determined the effect of Ntn-1 on intracellular ROS production. Ntn-1 elevated the level of ROS production (Fig. 3C), but importantly ROS production was abolished by a treatment of PP2 and the combination of the Inα6- and Inβ4-function-blocking antibodies, but not PP3 (Fig. 3D). The increased levels of ROS via Inα6β4 and c-Src were further visualized by staining the hUCB-MSC with a fluorescent dye, CM-H_2_DCFDA (Fig. 3E). Interestingly, a pre-treatment with *Rac1*siRNA or the lipid raft sequester methyl-β-cyclodextrin (MβCD) significantly blocked the ROS production induced by Ntn-1 (Fig. 3F). Moreover, cell proliferation by Ntn-1 was significantly blocked by a treatment with MβCD as well as an antioxidant, N-acetylcysteine (NAC) (Fig. 3G). These results demonstrate that lipid raft-mediated ROS production is a critical step in the regulation of cell proliferation induced by Ntn-1.

### Regulatory effect of Ntn-1 on SP1 activation and VEGF expression

**Figure 4:**
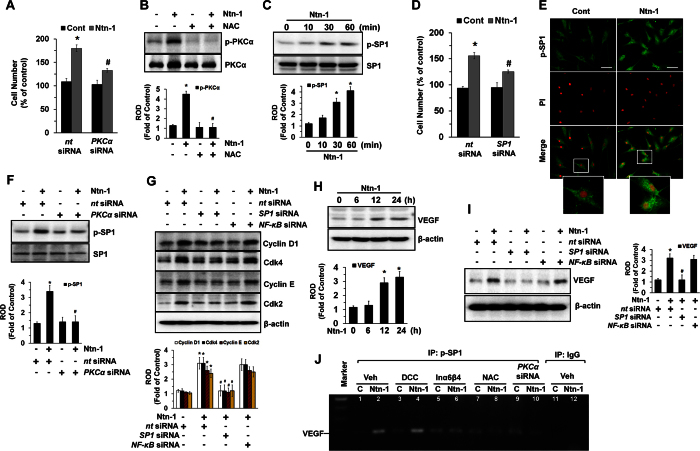
Regulatory effect of Ntn-1 on SP1 activation and VEGF expression. (**A**) The number of cells transfected with *PKC*αsiRNA prior to Ntn-1 exposure for 24 h is shown. Data represent the mean ± S.E. *n* = 3. *P < 0.05 vs. *nt* siRNA alone. ^*#*^*P* < 0.05 vs. *nt* siRNA + Ntn-1 alone. (**B**) Activation of PKCα in cells treated with NAC (10 μM) for 30 min prior to Ntn-1 exposure for 60 min is shown. Data represent the mean ± S.E. *n* = 3. *P < 0.01 vs. vehicle. ^*#*^*P* < 0.01 vs. Ntn-1 alone. (**C**) Phosphorylation of SP1 is shown. Data represent the mean ± S.E. *n* = 3. *P < 0.05 vs. 0 h. (**D**) The number of cells transfected with *SP1*siRNA prior to Ntn-1 exposure for 60 min is shown. (**E**) p-SP1 expression (green) was determined by confocal microscopy. Propidium iodide (PI) was used for nuclear counterstaining (red). Scale bars, 100 μm (magnification, x400). *n* = 3. (**F**) Phosphorylation of SP1 in cells transfected with *PKC*αsiRNA prior to Ntn-1 exposure for 60 min is shown. (**G**) The level of cell cycle proteins in cells transfected with *SP1*siRNA or *NF-κB*siRNA is shown. (**H**) The level of VEGF is shown. Data represent the mean ± S.E. *n* = 3. *P < 0.05 vs. 0 h. (**I**) The amount of VEGF in cells transfected with *SP1*siRNA or *NF-κB*siRNA prior to Ntn-1 exposure for 24 h is shown. (**J**) Cells were treated with DCC-function-blocking antibody, a combination of Inα6- and Inβ4-function-blocking antibodies, and NAC, or transfected with *PKC*αsiRNA prior to Ntn-1 exposure for 12 h. The binding of p-SP1 to *VEGF* promoter was determined by ChIP assay. *n* = 3. Normal mouse IgG was used as negative control for the ChIP. *n* = 3. (**D**,**F,G,I**) Data represent the mean ± S.E. *n* = 5. *P < 0.05 vs. *nt* siRNA. ^*#*^*P* < 0.05 vs. *nt* siRNA + Ntn-1. (**B**,**C**,**F**–**H**,**I**) ROD is the abbreviation for relative optical density.

ROS dominantly regulates PKC activation. Given that Ntn-1 specifically activates the PKCα isoform^11^, we further assessed whether PKCα activation is involved in cell proliferation. The knockdown of PKCα by *PKCα*siRNA inhibited cell proliferation as induced by Ntn-1 (Fig. 4A). Moreover, a pre-treatment with the antioxidant NAC significantly blocked PKCα activation (Fig. 4B). These results demonstrate that ROS production is linked to PKCα activation in promoting hUCB-MSC proliferation. We further examined the role of Ntn-1 in the activation of a transcription factor, SP1, as an important PKC signaling intermediate involved in stem cell proliferation^28^. Ntn-1 induced SP1 phosphorylation for 60 min (Fig. 4C). The knockdown of SP1 with *SP1*siRNA significantly blocked Ntn-1-induced cell proliferation (Fig. 4D). The increased accumulation of SP1 phosphorylation in the nucleus was further confirmed by immunofluorescence staining and counter-labeling with propidium iodide (PI) (Fig. 4E). Moreover, SP1 phosphorylation evoked by Ntn-1 was markedly attenuated by a treatment with *PKCα*siRNA (Fig. 4F), suggesting that PKCα-mediated SP1 activation is a key step in the promotion of hUCB-MSC proliferation as induced by Ntn-1. The knockdown of SP1 with *SP1*siRNA significantly decreased the levels of Cyclin D1, Cdk4, Cyclin E, and Cdk2 induced by Ntn-1 (Fig. 4G). Importantly, however, the knockdown of NF-κB, which is known to be required for Ntn-1-mediated cell migration^11^, did not have any effect on the level of cyclins and CDKs, indicating that the Ntn-1-mediated transition from the G_1_ to the S phase in stem cells is uniquely mediated by the phosphorylation of SP1 (Fig. 4G). In addition to cell proliferation, it is plausible that SP1 has the ability to regulate the gene expression activities involved in angiogenesis with regard to the regeneration of ischemic or injured tissues. We found that Ntn-1 significantly increased the amount of the vascular endothelial growth factor (VEGF) (Fig. 4H). The level of VEGF was decreased by a treatment with *SP1*siRNA, whereas the knockdown of *NF-κB* failed to regulate the protein level (Fig. 4I). These results indicate that SP1 is required for cell proliferation and for the angiogenic capacity of hUCB-MSC primed by Ntn-1. To determine whether SP1 directly regulates *VEGF* mRNA expression, we undertook a *VEGF* promoter region analysis using Alggen Promo^29^,^30^. The *VEGF* promoter contains five putative SP1 binding sites which are located between the −250 bp upstream site and the transcription start site. We performed a chromatin immunoprecipitation (ChIP) assay in hUCB-MSC treated with Ntn-1 to determine the regulatory effect of Ntn-1 on the binding of SP1 to the *VEGF* promoter. We tested a primer that includes five putative *VEGF* binding sites at the region proximal −2 bp to −248 bp of the start site of the *VEGF* promoter. We found that our primer sets result in an amplicon from the anti-pSP1 immunoprecipitates and importantly that the interaction of SP1 with the *VEGF* promoter was enhanced by the Ntn-1 treatment (Fig. 4J). Interestingly, however, the level of SP1 binding to the *VEGF* promoter was significantly inhibited by a pre-treatment with the combination of the Inα6- and Inβ4-function-blocking antibodies, NAC, and the knockdown of PKCα (Fig. 4J). These results suggest that Ntn-1 acting on Inα6β4 transcriptionally regulates SP1 binding to the *VEGF* promoter via lipid raft-mediated ROS production for cell proliferation.

### Regulatory effect of Ntn-1 on the F-actin reorganization of stem cells

**Figure 5:**
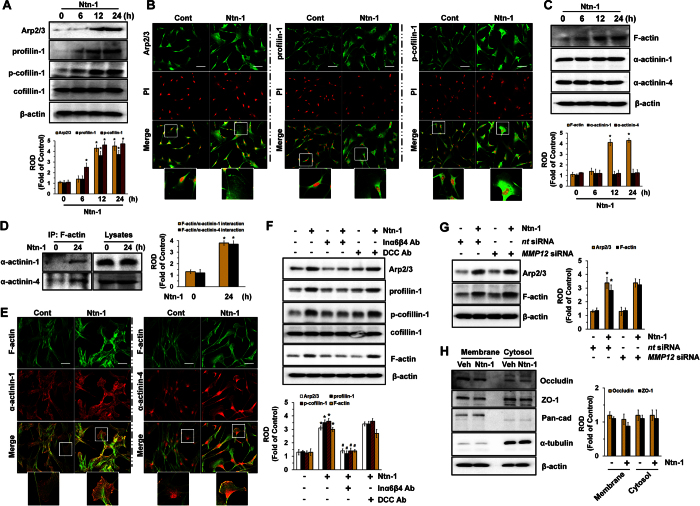
Regulatory effect of Ntn-1on F-actin reorganization of stem cells. (**A**) The level of Arp2/3, profilin-1, p-cofilin-1, and cofilin-1 in cells treated with Ntn-1 for 24 h is shown. Data represent the mean ± S.E. *n* = 4. *P < 0.01 vs. 0 h. (**B**) The increased expressions of Arp2/3 (green, left panel), profilin-1 (green, middle panel) and p-cofilin-1 (green, right panel) were determined by confocal microscopy. Propidium iodide (PI) was used for nuclear counterstaining (red). Scale bars, 100 μm (magnification, x400). *n* = 3. (**C**) Time responses of Ntn-1 in expression of F-actin, α-actinin-1, and α-actinin-4 for 24 h are shown. Data represent the mean ± S.E. *n* = 4. *P < 0.01 vs. 0 h. (**D**) Co-immunoprecipitation of F-actin with α-actinins is shown (left panel). The level of α-actinin-1 and −4 in total cell lysates is shown in the right panel. Data represent the mean ± S.E. *n* = 3. *P < 0.05 vs. 0 h. (**E**) The increased colocalization of F-actin (green) with α-actinin-1 and −4 (red) was determined by confocal microscopy. Scale bars, 100 μm (magnification, x400). *n* = 3. (**F**) The level of Arp2/3, profilin-1, p-cofilin-1, cofilin-1, and F-actin in cells pre-treated with DCC-function-blocking antibody and a combination of Inα6- and Inβ4-function-blocking antibodies for 30 min prior to Ntn-1 exposure for 24 h is shown. Data represent the mean ± S.E. *n* = 4. *P < 0.01 vs. vehicle. ^*#*^*P* < 0.01 vs. Ntn-1 alone. (**G**) The effect of Ntn-1 on the amount of Arp2/3 and F-actin in cells transfected with *MMP12*siRNA is shown. Data represent the mean ± S.E. *n* = 4. *P < 0.01 vs. *nt* siRNA. (**H**) The level of Occludin and ZO-1 presented in membrane and cytosol fraction in cells treated with Ntn-1 for 24 h was determined by Western blot analysis. Pan-cadherin and α-tubulin were used as internal controls for plasma membrane and cytosol, respectively. Data represent the mean ± S.E. *n* = 4. (**A**–**D**,**F**–**H**) ROD is the abbreviation for relative optical density.

We previously reported that Ntn-1 acts during the initiation of cytoskeletal reorganization in mesenchymal stem cells^11^ and in many other cell types^7^,^31^, but the underlying cellular mechanisms of Ntn-1 on cytoskeletal reorganization and the receptor specificity of stem cells involved in this process remain to be elucidated. An increase in Arp2/3, profilin-1, and p-cofilin-1 in response to Ntn-1, believed to be essential in the dynamic regulation of F-actin, was observed for 48 h at 50 ng/mL in a time-dependent manner (Fig. 5A). The increased level of proteins related to cytoskeletal reorganization as induced by an Ntn-1 treatment was further confirmed by immunofluorescence staining (Fig. 5B). A treatment with Ntn-1 also increased the amount of F-actin, but not the amounts of α-actinin-1 or α-actinin-4 (Fig. 5C). However, the F-actin interaction levels with α-actinin-1 and α-actinin-4 were increased after a treatment with Ntn-1 (Fig. 5D). Interestingly, the results according to confocal immunofluorescence microscopy revealed that Ntn-1 significantly induced the co-localization of F-actin and α-actinin-1 (Fig. 5E, left panel), as well as that of F-actin and α-actinin-4 (Fig. 5E, right panel). Importantly, the blocking of Inα6β4 activity but not DCC significantly attenuated the level of the cytoskeletal-reorganization-related proteins induced by Ntn-1 (Fig. 5F), whereas MMP-12 knockdown failed to regulate this process (Fig. 5G). Despite the frequent involvement of adherent junction molecules, such as E-cadherin, connexin, and occludin in the regulation of the reorganization of stem cells^32^,^33^, Ntn-1 did not have any effect on the protein amounts or locations of adherent junction molecules (Fig. 5H). We subsequently evaluated whether Ntn-1 distinctively regulates the Inα6β4 signaling pathway during cell proliferation and during the cytoskeletal reorganization of hUCB-MSC. We found that the levels of Arp2/3 and F-actin as induced by Ntn-1 were significantly attenuated by a pre-treatment with PP2 (Supplementary Fig. 2A), *Rac1*siRNA (Supplementary Fig. 2B), and MβCD (Supplementary Fig. 2C). Interestingly, the blocking of ROS production with NAC (Supplementary Fig. 2C) or the knockdown of PKCα with *PKCα*siRNA (Supplementary Fig. 2D) failed to regulate the cytoskeletal reorganization process of hUCB-MSC induced by Ntn-1, suggesting that Ntn-1 distinctively regulates the Inα6β4 signaling pathway, where the downstream signaling pathway of ROS production is necessary for the induction of hUCB-MSC proliferation.

### Effects of Ntn-1 on mouse skin wound healing and a hindlimb ischemia model

**Figure 6:**
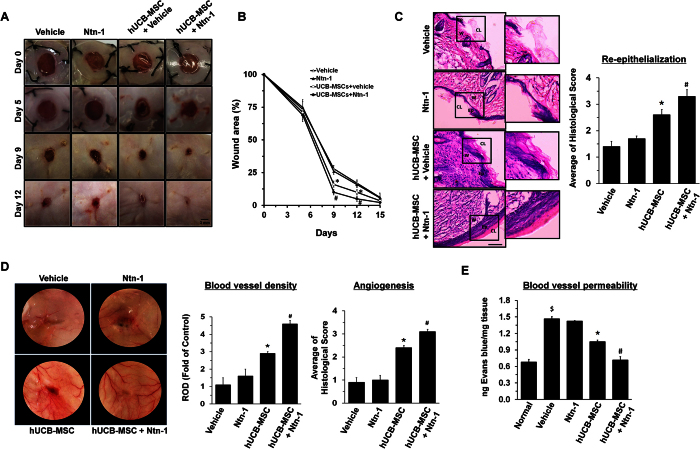
Effects of Ntn-1 on mouse skin wound healing. (**A**) Representative gross images on skin wound healing on day 0, 5, 9, and 12 are shown (left panel). Mouse skin wounds were made by 6-mm-diameter biopsy punch and treated with vehicle, Ntn-1, hUCB-MSC + vehicle, and hUCB-MSC + Ntn-1, respectively. (**B**) Quantifications of wound sizes relative to original wound size for 15 days are shown. Data represent the mean ± S.E. *n* = 7. *P < 0.05 vs. vehicle alone. ^*#*^*P* < 0.05 vs. hUCB-MSC + vehicle. (**C**) Representative wound tissues stained with H&E on day 12 are shown (Top panel). *n* = 7. Scale bars, 100 μm. Abbreviations: Ep, epidermis; W, wound bed; CL, cornified layer. Histological scores in re-epithelialization were quantified according to the Supplementary Table 1 (right panel). (**D**) Representative images of blood vessels in wounds on day 12. *n* = 7 (left panel). Vessel densities relative to the group treated with Vehicle alone were quantified by using Image J program (middle panel). ROD, relative optical density. Histological scores in angiogenesis were quantified according to the Supplementary Table 1 (right panel). (**E**) Upon postoperative day 12, Miles assay was performed to measure blood vessel permeability. Wound tissue samples (100 mg) were incubated with 500 μL formamide to release the extravagated Evans blue for 24 h. Optical density was measured at 610 nm and the measurements converted into ng dye extravagated per mg tissue (*n* = 7). ^$^*P* < 0.01 versus Normal. *P < 0.01 versus vehicle. ^#^*P* < 0.05 versus hUCB-MSCs alone.

To confirm the functional roles of Ntn-1 in promoting hUCB-MSC proliferation, we further explored the effect of hUCB-MSC pre-treated with 50 ng/mL of Ntn-1 on skin wound healing in mice. On days 9 and 12, the group given hUCB-MSC + Ntn-1 showed an increased extent of wound closure compared to the other groups (Fig. 6A,B). However, our data revealed there was no statistical difference between Ntn-1 alone and a vehicle, although spontaneous wound healing was observed. A histologic examination on day 12 showed that the wound bed was still not completely covered with epidermis in mice treated with the vehicle or Ntn-1 alone (Fig. 6C). However, the hUCB-MSC transplantation groups showed increased re-epithelialization from a mechanical skin wound, but the mice group that received hUCB-MSC + Ntn-1 showed enhanced levels of the wound closure, granulation, and re-epithelialization at mouse skin wound sites, resulting in nearly complete restoration of the epidermis. There were no additional differences between UCB-MSC + Ntn-1 and UCB-MSC on day 15. Histological scores for re-epithelialization and angiogenesis were quantified according to Supplementary Table 1. We found that the group given either hUCB-MSC + Ntn-1 or hUCB-MSC showed significantly augmented levels of re-epithelialization compared to the group treated with the vehicle or Ntn-1 alone (Fig. 6C, bottom panel). In addition, the transplantation of hUCB-MSC + Ntn-1 showed an enhanced level of epidermal organization compared to that by hUCB-MSC alone. Consistently, our results revealed that the group given hUCB-MSC + Ntn-1 showed significantly increased blood vessel density and angiogenesis, with the number of blood vessels toward the wound surface greater than that by hUCB-MSC (Fig. 6D). There were no statistical differences between the groups treated with Ntn-1 alone and the vehicle in terms of wound healing and angiogenic capacity. We performed an additional experiment to assess the degree of blood vessel permeability using a Miles assay, measuring the amount of the extravasation of Evans blue. As shown in Fig. 6E, our results revealed that the level of Evans blue extravasation in mice given hUCB-MSC + Ntn-1 was significantly lower than that by hUCB-MSC alone, resulting in the nearly complete restoration of blood vessel permeability. There were no significant differences between the blood vessel permeability of mice treated with a vehicle or with Ntn-1 alone.

**Figure 7:**
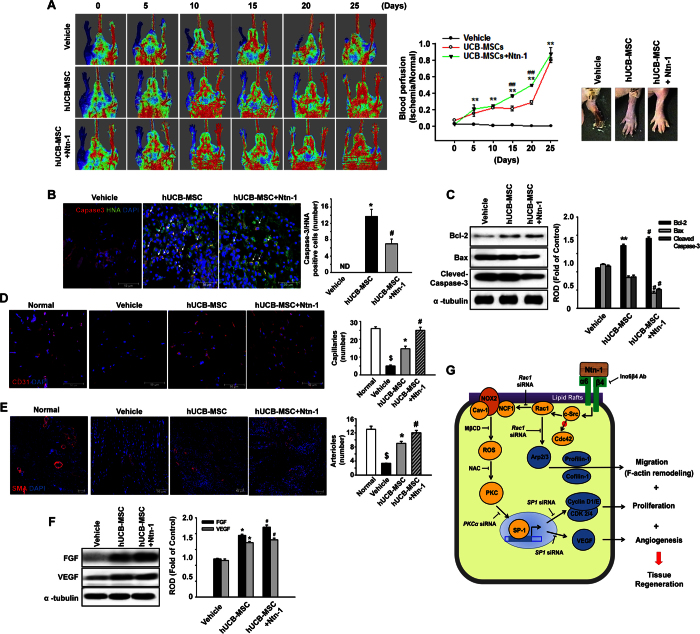
Effects of Ntn-1 on vascular regeneration in mouse hindlimb ischemia model. (**A**) The ratio of blood perfusion (blood flow in the left ischemic limb/blood flow in the right non-ischemic limb) was measured using Laser Doppler perfusion imaging analysis in the ischemic limbs of nude mice injected with PBS, hUCB-MSC, or hUCB-MSC + Ntn-1 on postoperative days 0, 5, 10, 15, 20, and 25 (left and middle panels). Gross morphologies of mice hindlimb on postoperative day 15 are shown (right panel). Data represent the mean ± SE. *n* = 5. **P < 0.01 vs. vehicle, ^*##*^*P *<* *0.01 vs. hUCB-MSC + Ntn-1. (**B**) The survival of transplanted hUCB-MSCs was assessed by immunofluorescent staining with Cleaved Caspase-3 (red) and anti-human nuclei antigen (HNA, green) antibodies. DAPI was used as nuclear control (blue). *n* = 3. Scale bar, 50 μm. Apoptotic cells were quantified as the number of HNA- and Cleaved Caspase-3-positive cells. (**C**) The level of apoptosis-related proteins in the mice treated with vehicle, hUCB-MSCs, and hUCB-MSCs + Ntn-1 on 3 days was determined by Western blot with Bcl-2, Bax, and Cleaved Caspase-3 antibodies. *n* = 3. *P < 0.01 versus vehicle. ^*#*^*P* < 0.05 versus hUCB-MSCs alone. Data represent the means ± S.E. Upon postoperative day 15, ischemic limb tissue samples were immunostained with an anti-CD31 (**D**) and anti-α-SMA (**E**) for assessment of capillary density and arteriole density, respectively. *n* = 3. Scale bar, 50 μm. The density of capillary and arteriole was quantified as the number of CD31- and α-SMA-positive cells. ^$^*P* < 0.01 versus Normal. *P < 0.01 versus vehicle. ^*#*^*P* < 0.05 versus hUCB-MSCs alone. (**F**) The level of FGF and VEGF in the mice treated with vehicle, hUCB-MSCs, and hUCB-MSCs + Ntn-1 for 15 days was determined by Western blot. *P < 0.01 versus vehicle. ^*#*^*P* < 0.05 versus hUCB-MSCs alone. Data represent the means ± S.E. n = 3. (**G**) A hypothetical model for Ntn-1-induced signaling pathway in promoting mouse wound healing and vascular regeneration. (**C**,**F**) ROD is the abbreviation for relative optical density.

We also attempted to determine the angiogenic capacity of Ntn-1 on hindlimb ischemia in the mouse model using a laser Doppler imager (Fig. 7A). Upon postoperative day 25, progressive recoveries of blood perfusion and the nearly complete restoration of the hindlimb were observed in the hUCB-MSC transplantation groups. However, the group given hUCB-MSC + Ntn-1 showed an enhanced ischemic/normal hindlimb blood perfusion ratio on postoperative days 15 and 20 compared to the ratio with hUCB-MSC alone. There were no significant differences between the blood perfusion ratio of mice treated with a vehicle or with 50 ng/mL of Ntn-1 alone (data not shown). Upon postoperative day 3, importantly, Ntn-1 significantly reduced the number of apoptotic cells corresponding to the transplanted hUCB-MSC (Fig. 7B). Consistently, the group given hUCB-MSC + Ntn-1 showed an increased Bcl-2 amount but decreased levels of Bax as well as cleaved-caspase-3 compared to the results by hUCB-MSC alone, suggesting that Ntn-1 enhances the survival of transplanted hUCB-MSCs in ischemic tissues (Fig. 7C). To evaluate the role of hUCB-MSC pre-treated with Ntn-1 in neovascularization, CD31-positive capillaries and α-SMA-positive arterioles were quantified in ischemic tissues by immunohistochemistry on postoperative day 15. The results of immunofluorescent staining for CD31 (Fig. 7D) and α-SMA (Fig. 7E) revealed that the number of capillaries and arterioles were significantly increased in the group that received hUCB-MSC + Ntn-1 compared to that by hUCB-MSC alone. Moreover, hUCB-MSCs activated by Ntn-1 were shown to enhance the levels of FGF and VEGF respectively, compared to the results by hUCB-MSC alone (Fig. 7F). These results indicate that the pre-activation of hUCB-MSC with Ntn-1 facilitates vascular regeneration in ischemic tissues.

## Discussion

In the present study we showed that Ntn-1 has the ability to induce stem cell proliferation via the distinct lipid raft-dependent Inα6β4 signaling pathway, which plays a critical role in the governing of VEGF induction and the cytoskeletal reorganization of hUCB-MSC. Our data demonstrate that Ntn-1-mediated hUCB-MSC bioactivity improves the regeneration process of injured tissues. Concerning the cellular mechanisms of Ntn-1 with regard to amplifying the level of stem cell bioactivity, we showed a unique relationship between the lipid raft-dependent Inα6β4 signaling pathway and ROS production in the regulation of hUCB-MSC proliferation. It was shown that hUCB-MSCs express DCC and Inα6β4 as key functional receptors of Ntn-1 for stem cell survival and motility^11^,^12^, but the expression levels of UNC5A, UNC5B, UNC5C, or neogenin were very low in these cells. In the present study, however, DCC was located on the non-lipid raft part and did not influence hUCB-MSC proliferation. Instead, Ntn-1 induced stem cell proliferation via the distinct lipid raft-dependent Inα6β4 signaling pathway. Indeed, it was previously demonstrated that Ntn-1 mediates chemotropic guidance of nerve growth cones via lipid raft-dependent signaling pathway^27^. Our findings are further supported by a previous study in which Inα6β4 was found to act as a link between the cytoskeleton and extracellular matrix molecules to initiate a variety of intracellular signalization processes, including cell proliferation, VEGF expression, and cytoskeletal reorganization in cooperation with growth factor receptors^34^. To the best of our knowledge, this is the first study to provide evidence that Ntn-1 distinctively regulates the bioactivity of stem cells via the Inα6β4 signaling pathway. On the other hand, our data indicate that Ntn-1 acting through Inα6β4 induces cell proliferation via c-Src phosphorylation. An earlier study showed that c-Src is a key immediate downstream effector of Inα6β4 and that its activity is required for Inα6β4 signaling competency in promoting cell proliferation^35^,^36^ and the growth factor signaling pathway^37^. Hence, it is plausible that Ntn-1 coupled with Inα6β4 is an important signaling cue to trigger c-Src activation. Consistently, we provided compelling evidence that Ntn-1 uniquely activates Rac1 via c-Src activation in promoting hUCB-MSC proliferation. In support of our data, it was reported that active c-Src can phosphorylate and potentiate Tiam1, a guanine nucleotide exchange factor (GEF) which exhibits the highest specificity for Rac1, indicating that Rac1 is mainly activated by c-Src via Tiam1^38^. Hence, our results are consistent with the notion that Ntn-1 stimulates the Inα6β4-meidated c-Src signaling pathway and that c-Src is required for the distinct regulation of Rac1 in promoting hUCB-MSC proliferation.

The important finding is that Ntn-1 induces Rac1 clustering with NADPH oxidases in the lipid raft for ROS production. Increasing evidence has suggested that lipid rafts are clustered to form a redox signaling platform through NOX2 coupling with cytosolic factors that include NCF1, p67 ^phox^, and Rac1 and that these processes subsequently produce superoxides and other ROS^39^,^40^. Although in most cell types, mitochondrial ROS are thought to be the largest contributor to intracellular ROS production^41^, our results revealed that the sequestration of cholesterol attenuates ROS production and hUCB-MSC proliferation as induced by Ntn-1. Hence, this suggests that epithelial ROS generated by NADPH oxidase within lipid rafts may influence cell proliferation. Subsequently, our data revealed that Ntn-1 induces PKCα activation via ROS production. ROS is a major mediator in the amplification of the PKC signaling pathway via the regulation of phospholipase C and Ca^2+^ concentrations, which are all related to cell proliferation during the neuronal development process induced by Ntn-1^7^,^42^,^43^,^44^. Thus, these results suggest that PKCα primed by ROS plays an important role as a signal messenger in promoting cell proliferation via the lipid raft-mediated Inα6β4 signaling pathway. In an attempt to uncover the mechanism by which PKCα induces hUCB-MSC proliferation, our data revealed that SP1 phosphorylation is involved in this process. Moreover, Ntn-1 activated SP1 to elevate the level of cyclin D1/E and its catalytic partner Cdk4/2, which are known to play important roles in the G_1_/S checkpoint of the cell cycle. This SP1 activation step was unique to cell proliferation, as NF-κB failed to regulate cell cycle progression in response to Ntn-1. Congruently, an earlier study showed that PKCα regulates the nuclear location and activity of MAPKs to stimulate SP1 phosphorylation in promoting the proliferation of many types of cells^11^,^45^,^46^. Together, these results indicate that SP1 is a key regulator of the Ntn-1signaling pathway to regulate hUCB-MSC proliferation. In addition to cell proliferation, our results indicate that Ntn-1-induced SP1 activation influences the expression of VEGF as well. These findings mean that SP1 is the relevant target as a link between Ntn-1-elicited proliferative and angiogenic responses. Earlier results in agreement with these findings also showed that high GC-rich motifs in regions close to the VEGF promoter are regulated by SP1, thereby regulating basal VEGF expression levels^45^,^47^,^48^. Thus these results suggest that Ntn-1 enhances the transcriptional targeting of SP1 for close cooperation with the cell proliferation machinery and angiogenic capacity to amplify hUCB-MSC bioactivity.

In addition to the role of Ntn-1 to modulate hUCB-MSC proliferation and VEGF expression, our results indicate a potential role of Ntn-1 in the regulation of the cytoskeletal reorganization process. We found that the Ntn-1 signaling pathway is crucially linked to Rac1/Arp2/3 activation to promote proteins related to cytoskeletal reorganization. Our data are also supported by previous work showing that integrin receptor activation acting on Rac1 stimulates actin cytoskeleton reorganization by recruiting the Arp2/3 complex that bypasses the N-WASP function^49^. It was clearly shown that profilin-1 and cofilin-1 play key roles in enhancing the actin assembly at the plasma membrane, thereby increasing F-actin expression, which drives cell motility and other actin-linked processes^50^,^51^. Specifically, α-actinin cross-links F-actin into bundles to form a cytoskeletal network^52^. Consistent with this, our results revealed that F-actin/α-actinin-1 and F-actin/α-actinin-4 interactions are increased by an Ntn-1 treatment. Although the exact mechanism by which netrin receptors and signal-transduction proteins participate in cytoskeletal reorganization remains to be determined, we have proven that the ROS-mediated PKC pathway is not involved in this mechanism, suggesting that Ntn-1 uniquely regulates cytoskeletal reorganization via the Inα6β4 signaling pathway.

Finally, we showed convincing *in vivo* proof that the transplantation of hUCB-MSCs pre-treated with Ntn-1 accelerates skin wound healing, although Ntn-1 by itself did not have the ability to aid this process. Moreover, we observed that hUCB-MSCs activated by Ntn-1 facilitate vascular regeneration by improving the angiogenic capacities. Thus, hUCB-MSCs may be more useful if they are pre-activated by Ntn-1, as doing so can demonstrate the potential benefits of stem cell transplantation therapy for tissue regeneration not only with timely efficacy, but also with a reduction of the side effect of an overdose of Ntn-1. In addition, the pre-activation of hUCB-MSCs with Ntn-1 may offer a means to improve the potency of these cells without the need for additional cell numbers. The majority of mesenchymal stem cells have been shown to enhance the regeneration of damaged tissues via a paracrine mechanism rather than through multi-lineage differentiation^1^,^53^. Thus, it is possible that the transplantation of hUCB-MSC pre-treated with Ntn-1 enhances paracrine mechanisms to promote the regeneration of ischemic or injured tissues. Our results are consistent with the notion that MSCs treated with TNF-α stimulate the tissue regeneration process through paracrine mechanisms^53^. It was also noted that UCB-MSC + Ntn-1 transiently enhances skin wound healing and blood flow recovery on days 9–12 and days 15–20, respectively. Thus, we anticipate that a time of 3~5 days represents the maximum duration for Ntn-1 to have transient effects on skin wound healing and vesicular regeneration in the mouse model. The time discrepancy with regard to the functional role of Ntn-1 between skin wound healing and vesicular regeneration may be due to differences in the mouse strain, animal model, wound location and size, and/or the experimental conditions. Thus, we also anticipate that Ntn-1 stimulates hUCB-MSC proliferation and F-actin reorganization at wound sites, where the improved potency of hUCB-MSCs brings their transient functional effects forward during the skin wound healing process compared to vesicular regeneration through either a sequential result of the activation of different types of cells in the wound area or, alternatively, an independent process involving other cellular signaling events. On the other hand, a previous report showed that the direct co-treatment of Ntn-1 with rat bone-marrow-derived MSCs onto muscle facilitates revascularization in a hindlimb ischemia model, although the level of the Ntn-1 concentration was extremely high (>1 mg/mL)^14^. However, it was proved that Ntn-1 concentrations exceeding 1 μg/mL may not induce endothelial cell migration and may even prevent endothelial cell movement^54^. Such biphasic effects have also been observed with other angiogenic molecules, such as angiopoietin, endostatin, or thrombospondin-1^55^. Given the physiological concentration ranges of 50 to 150 ng/mL that are required for axon outgrowth as well as angiogenesis^56^, our results here indicate that Ntn-1 at physiological levels (50 ng/mL) has the ability to amplify the bioactivity of stem cells and that the pre-activation of the hUCB-MSC with 50 ng/mL of Ntn-1 improves the regeneration of ischemic or injured tissues. Consistently, previous reports showed that Ntn-1 at 50 ng/mL has the ability to stimulate the survival and motility of stem cells^11^,^12^. Therefore, our findings suggest that Ntn-1 is a good candidate for stem cell transplantation as a pre-activation agent.

Taken together, our findings suggest that Ntn-1 plays an important role in improving the bioactivity of hUCB-MSC via the distinct lipid raft-mediated Inα6β4 signaling pathway, which is critical for providing a suitable microenvironment for hUCB-MSC transplantation and tissue regeneration (Fig. 7G). In conclusion, the Ntn-1/Inα6β4 axis triggers the phosphorylation of c-Src to regulate Rac1 activation in the membrane lipid raft part, where Ntn-1 uniquely regulates ROS production to facilitate the PKC-mediated SP1 signaling pathway.

## Materials and Methods

### Materials

Human umbilical cord blood derived mesenchymal stem cells (hUCB-MSC) were kindly provided by Medipost Co. (Seoul, Korea), which was isolated and expanded as reported previously^18^. These cells have been characterized to express CD105 (99.6%) and CD73 (96.3%), but not CD34 (0.1%), CD45 (0.2%) and CD14 (0.1%). They were positive for HLA-AB but generally not for HLA-DR^18^. The human UCB-derived MSCs differentiated into various cell types such as osteoblasts, chondrocytes, and adipocytes upon *in vitro* induction with the appropriate osteogenic, chondrogenic, and adipogenic differentiation stimuli^18^. The following antibodies were purchased: Cyclin D1, Cyclin E, CDK2, CDK4, F-actin, and p-PKCα^Ser657^ antibodies (Cell Signaling Technology, Danvers, MA, USA); Cdc42, NOX2, Rac1, and RhoA antibodies (BD Biosciences, Franklin Lakes, NJ, USA); mouse monoclonal DCC antibody (Calbiochem, La Jolla, CA, USA), NCF-1 antibody (LifeSpan Biosciences, Seattle, WA, USA); α-actinin-1, α-actinin-4, Arp2/3, β-actin, rabbit polyclonal Inα6, rabbit polyclonal Inβ4, MMP-12, p-NF-κBp65^Ser536^, NF-κB, pan-cadherin, p-cofilin-1^Ser3^, cofilin-1, profilin-1, SP1, p-SP1^Thr453^, c-Src, p-c-Src^Tyr419^, and VEGF antibodies (Santa Cruz Biotechnology, Paso Robles, CA, USA); Horseradish peroxidase (HRP)-conjugated goat anti-rabbit and goat anti-mouse IgG antibodies (Jackson Immunoresearch, West Grove, PA, USA). 2′, 7′-dichlorofluorescein diacetate (CM-H_2_DCFDA) was obtained from Invitrogen (Carlsbad, CA, USA). Methyl-β-cyclodextrin (MβCD), N-Acetyl-l-cysteine (NAC), PP2, PP3, and formamide were purchased from Sigma Chemical Company (St. Louis, MO, USA). Netrin-1 (Ntn-1) was obtained from R&D Systems (Minneapolis, MN, USA). All of the pharmacological inhibitors listed did not show any significant cytotoxic effects by themselves as confirmed by FACS analysis in each experiment. All other reagents were of the highest purity commercially available and were used as received.

### Culture of hUCB-MSCs

hUCB-MSCs were cultured without a feeder layer in the α-minimum essential medium (α-MEM; Thermo, MA, USA). The cells were grown in 1% penicillin and streptomycin, and 10% FBS. For each experiment, cells were grown in wells of 6- and 12-well plates, and in 35, 60, or 100-mm diameter culture dishes in an incubator maintained at 37 °C with 5% CO_2_. The medium was replaced with serum-free α-MEM at least 24 h before experiments. Following incubation, the cells were washed twice with phosphate-buffered saline (PBS) and then maintained in a serum-free α-MEM including all supplements and indicated agents.

### Mouse skin wound healing model

All animal procedures were performed following the National Institutes of Health Guidelines for the Humane Treatment of Animals, with approval from the Institutional Animal Care and Use Committee of Seoul National University (SNU-140123-6). Eight week-old male ICR mice were used. All surgery was performed under anesthesia using a 2:1 mixture of Zoletil^TM^ (20 mg/kg, Virbac Laboratories, Carros, France) and Xylazine HCl (10 mg/kg, Rompun^®^, Bayer, Germany) and all efforts were made to minimize suffering. In addition, six authors were Doctors of Veterinary Medicine with licenses granted from the Ministry of Agriculture and Forestry of Republic of Korea. Mouse skin wounding and stem cell implantation were performed as described previously^57^,^58^. Briefly, after shaving backs and scrubbing with an organic iodine solution, circular full-thickness wound was surgically created by using a 6-mm-diameter sterile biopsy punch. hUCB-MSCs were pre-treated with 50 ng/mL Ntn-1 for 24 h prior to the skin transplantation. To know the functional role of hUCB-MSCs pre-treated with Ntn-1, experimental animals were divided into four groups: wild type mice that were received vehicle (group 1, n = 7) or 50 ng/mL Ntn-1 (group 2, n = 7) without hUCB-MSCs; and hUCB-MSCs transplantation group mice that were given hUCB-MSCs pre-treated with vehicle (group 3, n = 7) or 50 ng/mL Ntn-1 (group 4, n = 7). We injected 1 × 10^6^ UCB-MSCs in 70 μL PBS containing 50% growth factor–reduced Matrigel (BD Biosciences, NJ, USA) into the dermis at two sites around the wound and topically applied 0.3 × 10^6^ UCB-MSCs in 30 μL PBS containing the 50% Matrigel onto the wound bed at day 0 and 9. After that, the wounds were dressed with Tegaderm (3 M, London, Canada). Images of wounds were made on days 0, 5, 9 and 12 with a digital camera system (D50, Nikon, Tokyo, Japan) at the same camera/subject distance (30 cm). The sizes of wound closure were determined by measuring wound resealing from the images captured at the wounded sites. The wound areas were measured planimetrically using the free-hand tool in Image J software (NIH, Bethesda, MD). Percent wound closure was calculated as the difference in wound size on a particular day compared to day 0 (time of wounding)/initial wound size. At day 12, the wound tissues were embed in O.C.T. compound (Sakura Finetek, CA, USA), stored at −70 °C, cut the samples to 6 μm thick frozen sections by using cryosectioning machine, and mounted on SuperFrost Plus slides (Thermo Fisher Scientific, IL, USA) for hematoxylin and eosin (H&E) staining and immunohistochemistry. The quantitative aspects of the score were evaluated by the percentage of the tissue presenting the specific qualitative features in re-epithelialization and angiogenesis during skin wound healing according to the method of Rajabi *et al.* (2007)^59^ (Supplementary Table 1) validated previously through similar experimental models^60^,^61^, and then the averages of the sum of scores for each mouse were calculated. Histological analysis was carried out by four independent observers who were unaware of the treatment.

### Miles assay

Mouse skin wounding experiments were performed as described above. Upon postoperative day 12, Miles assay was further performed to measure blood vessel permeability according to the method of Radu and Chernoff (2013)^62^. Mice (n = 7) were injected intravenously with 200 μL of 0.5% Evans blue solution for 30 min. Wound tissue samples (100 mg) were incubated with 500 μL formamide to release the extravagated dye. Tissue samples were incubated for 24 h at 55 °C water bath. The samples were then centrifuged at 14,000 × g for 10 min and 100 μL of supernatant quantified spectrophotometrically against a formamide blank at 630 nm. The absolute amount of dye was determined using a standard curve.

### Mouse hindlimb ischemia model

Experiments were performed on male 8-week-old Balb/C nude mice maintained under a 12 h light/dark cycle and in accordance with the regulations of Soonchunhyang University, Seoul Hospital. Animal procedures were approved by the guidelines of the Institutional Animal Care and Use Committee of Soonchunhyang University (IACUC2013-5). A hindlimb ischemia model was used as we described previously^63^. Ischemia was induced by ligation of the proximal femoral artery and boundary vessels of the mice. No later than 6 h after surgery, vehicle PBS (n = 5), 50 ng/mL Ntn-1, hUCB-MSCs (n = 5), or hUCB-MSCs pre-treated with 50 ng/mL Ntn-1 (n = 5) were injected into multiple (five) ischemic sites. Blood perfusion was assessed by measuring the ratio of blood flow in the ischemic (left) limb to that in the non-ischemic (right) limb on postoperative days 0, 5, 10, 15, 20, and 25 using Laser Doppler perfusion imaging (LDPI; Moor Instruments, Wilmington, DE). The ischemic thigh areas were removed at 3, 15, or 25 days post-hUCB-MSC transplantation for immunohistochemistry.

### Immunohistochemistry

The ischemic thigh areas were removed at 3, 15, or 25 days post-hUCB-MSC transplantation and fixed with 4% paraformaldehyde (PFA; Affymetrix, Santa Clara, CA). Each tissue sample was embedded in paraffin. Immunofluorescent staining was performed using primary antibodies against CD31, α-smooth muscle actin (SMA), cleaved-caspase-3 (Santa Cruz Biotechnology, Paso Robles, CA, USA), and human nuclear antigen (HNA, EMD Millipore, Billerica, MA) and secondary antibodies conjugated to Alexa488 and Alexa594 (Invitrogen, Carlsbad, CA, USA) in the dark case. Nuclei were stained with 4′,6-diaminido-2-phenylindol (DAPI; Vector Laboratories, Burlingame, CA). Immunostained slides were imaged by confocal microscopy (Olympus).

### Flow cytometry

Cells were synchronized in the G_0_/G_1_ phase by culture in serum-free media for 24 h before incubation of Ntn-1. The cells were then fixed with 70% ice-cold ethanol for 30 min at 4 °C, followed by incubation in a freshly prepared nuclei staining buffer consisting of 250 μg/mL propidium iodide (PI) and 100 μg/mL RNase for 30 min at 37 °C. The sample was read by flow cytometry and analyzed using CXP software (Beckman Coulter, Brea, CA).

### Cell number count

To determine total cell numbers, cells were washed twice with PBS and trypsinized from the culture dishes. The cell suspension was mixed with a 0.4% (w/v) trypan blue solution, and the number of live cells was determined using a hemocytometer. Cells failing to exclude the dye were considered nonviable.

### Small interfering (si)RNA transfection

Cells were grown until 75% of the surface of the plate and transfected for 36 h with ON-TARGETplus siRNAs mixed by 4 different siRNAs specific for MMP12, Rac1, CDC42, PKCα, NF-κBp65, or SP1 (GE Dharmacon, Lafayette, CO, USA) or non-targeting (*nt*) siRNA as a negative control (GE Dharmacon, Lafayette, CO, USA) with HiPerFect Transfection Reagent (QIAGEN, Valencia, CA, USA) according to the manufacturer’s instructions. The siRNA efficacies were determined by Western blot (Supplementary Fig. 3).

### Subcellular fractionation

Harvested cell pellets were mixed with buffer 1 (250 mM sucrose, 50 mM Tris-HCl, 5 mM MgCl_2_) in the presence of protease inhibitor cocktail (PIERCE, Rockford, IL, USA) and incubated for 10 min on an end-over-end shaker and centrifuged at 1,000 × g for 10 min. The supernatants with cytosolic protein were transferred to iced tubes. The pellet was suspended in buffer 2 (1 M sucrose, 50 mM Tris-HCl, 5 mM MgCl_2_) for 30 min and centrifuged at 6,000 × g for 10 min and the supernatants containing membrane proteins were transferred to new tubes. The remaining pellet was suspended in buffer 3 (20 mM Tris-HCl, 0.4 M NaCl, 15% glycerol, 1.5% Triton X-100) with protease inhibitor cocktail and incubated for 10 min on an end-over-end shaker. After centrifugation at 6,800 × g for 10 min, the supernatants were collected and designed as the nuclear proteins.

### Immunoprecipitation and Western blot analysis

Interaction of Rac1 with NOX2, NCF1, and caveolin-1, or F-actin with α-actinin-1 and -4 was analyzed by immunoprecipitation and Western blotting. Cells were lysed with lysis buffer (1% Triton X-100 in 50 mM Tris–HCl pH 7.4 containing 150 mM NaCl, 5 mM EDTA, 2 mM Na_3_VO_4_, 2.5 mM Na_4_PO_7_, 100 mM NaF, 200 nM microcystin lysine–arginine, and protease inhibitors). Cell lysates (400 μg) were mixed with 10 μg of each antibodies. The samples were incubated for 4 h, mixed with Protein A/G PLUS-agarose immunoprecipitation reagent (Pierce, Rockford, IL, USA) and then incubated for an additional 12 h. The beads were washed four times, and the bound proteins were released from the beads by boiling in SDS-PAGE sample buffer for 5 min. Samples were analyzed by Western blotting, which was performed as previously described with minor modifications^64^.

### Affinity precipitation of cellular GTP-Cdc42, -Rac1, and -RhoA

Activation of Cdc42, Rac1, and RhoA activities were determined by using an affinity precipitation assay kits (EMD Millipore, Billerica, MA, USA) according to the manufacturer’s instructions. Cells starved for 24 h were stimulated with Ntn-1 and lysed for 5 min in ice-cold cell lysis buffer. Four hundred micrograms of lysates were incubated for 1 h with agarose beads coupled with the Cdc42/Rac binding domain (GST-PAK-PBD) or with Rho-binding domain of rhotekin (GST-Rhotekin-RBD), and the bound Cdc42, Rac1, and RhoA proteins were eluted with 2 X laemmli sample buffer and subjected to Western blot using anti-Cdc42, anti-Rac1, and anti-RhoA antibodies, respectively.

### Detergent-free purification of caveolin-rich membrane fraction

**C**ells were washed twice with ice-cold PBS, scraped into 2 mL of 500 mM sodium carbonate (pH 11.0), transferred to a plastic tube, and homogenized with a Sonicator 250 apparatus (Branson Ultrasonic, Danbury, CT) using three 20-sec bursts. The homogenate was adjusted to 45% sucrose by the addition of 2 mL 90% sucrose prepared in 2-(N-morpholino) ethanesulfonic acid (MES)-buffered solution consisting of 25 mM MES-buffer solution (pH 6.5) and 0.15 M NaCl and placed at the bottom of an ultracentrifuge tube. A 5–35% discontinuous sucrose gradient was formed (4 mL each of 5% and 35% sucrose, both in MES-buffer solution containing 250 mM sodium carbonate) and centrifuged at 40,000 × g for 20 h in a Beckman SW41 Rotor (Beckman Coulter, Fullerton, CA). Eight fractions were collected and analyzed by 12% SDS-PAGE.

### Intracellular reactive oxygen species (ROS) detection

2′, 7′-dichlorofluorescein diacetate (CM-H_2_DCFDA) was used to detect the general ROS production. To quantify the intracellular ROS levels, the cells treated with 10 mM DCF-DA were rinsed twice with ice-cold PBS and then scraped. A 100 μL cell suspension was loaded into a 96-well plate and examined using a luminometer (Victor3; Perkin-Elmer, MA, USA) and a fluorescent plate reader at excitation and emission wavelengths of 485 and 535 nm, respectively.

### Immunofluorescence analysis

Cells were fixed in 4% paraformaldehyde in PBS for 10 min at room temperature, permeabilized in 0.1% Triton X-100 in PBS for 5 min, and blocked in PBS containing 5% (v/v) normal goat serum (NGS) for 30 min at room temperature. Samples were then stained with primary antibody for overnight at 4 °C. Following three washes with PBS, the samples were incubated with Alexa 488-conjugated goat anti-rabbit/mouse IgM (Invitrogen Co., Carlsbad, CA, USA), and counterstained with PI in PBS containing 5% (v/v) NGS for 2 h. After washing with PBS, samples were mounted on slides and visualized with an Olympus FluoView™ 300 confocal microscope with 400x objective.

### Chromatin Immunoprecipitation (ChIP)

ChIP was performed using the EZ-ChIP kit (EMD Millipore, Billerica, MA, USA) according to the manufacturer’s protocol. Briefly, cells were treated with 1% formaldehyde for 15 min to cross-link proteins to DNA, lysed, and then sonicated. The lysate was incubated with primary antibodies overnight at 4 °C. The immunocomplex was purified by incubation with 60 μL of protein G-agarose beads for 1 h and eluted for DNA purification. Quantitative real-time PCR was performed with primers for the *VEGF* promoter flanking the putative SP1 binding sites. The following primer sequences of *VEGF* promoter were used (sence and antisence respectively): −2 bp ~ −248 bp, 5′-TTTTCAGGCTGTGAACCTTG-3′ and 5′-GATCCTCCCCGCTACCAG-3′. Normal mouse IgG was used as the negative control for immunoprecipitation. The human *VEGF* promoter sequence was found using the Eukaryotic Promoter Database. The putative binding sites were predicted using Alggen Promo software, version 3.0.2^29^,^30^.

### Statistical analysis

Results are expressed as means ± standard errors (S.E.). All experiments were analyzed by ANOVA, followed in some cases by a comparison of treatment means with a control using the Bonferroni-Dunn test. Differences were considered statistically significant at *P* < 0.05.

## Additional Information

**How to cite this article**: Lee, S. S. *et al.* Netrin-1-Induced Stem Cell Bioactivity Contributes to the Regeneration of Injured Tissues via the Lipid Raft-Dependent Integrin α6β4 Signaling Pathway. *Sci. Rep.* **6**, 37526; doi: 10.1038/srep37526 (2016).

**Publisher's note:** Springer Nature remains neutral with regard to jurisdictional claims in published maps and institutional affiliations.

## Supplementary Material

Supplementary Information
