# Supplementary material for: Netrin-1-Induced Stem Cell Bioactivity Contributes to the Regeneration of Injured Tissues via the Lipid Raft-Dependent Integrin α6β4 Signaling Pathway
Source: Sci Rep. 2016 Nov 24;6:37526. doi: 10.1038/srep37526 (PMC5121594; doi:10.1038/srep37526)
Supplement: Supplementary Information [file srep37526-s1.pdf]

Supplementary information

Netrin-1-Induced Stem Cell Bioactivity Contributes to the Regeneration of Injured Tissues via the Lipid Raft-Dependent Integrin α6β4 Signaling Pathway

Soo Sang Lee[†, 1], Sei-Jung Lee[*, 1], Sang Hun Lee[§], Jung Min Ryu[¶], Hyeon Su Lim*,
Jun Sung Kim*, Eun Ju Song*,Young Hyun Jung*, Hyun Jik Lee*, Chung Hun Kim[†], Ho Jae Han*[‡]

Supplementary Table 1. Scoring of histological changes in wound healing

Score	Re-epithelialization	Angiogenesis
**0**	Absence of epithelial proliferation in > 70% of the tissue	Absence of angiogensis, edema, hemorrhage, congestion
**1**	Poor epidermal organization in > 60% of the tissue	1 - 2 vessels per site, edema, hemorrhage, congestion
**2**	Incomplete epidermal organization in > 40% of the tissue	3 - 4 vessels per site, moderate edema and congestion
**3**	Moderate epithelial proliferation in > 60% of the tissue	5 - 6 vessel per site, slight edema and congestion
**4**	Complete epidermal remodeling in > 80% of the tissue	More than 7 vessel per site

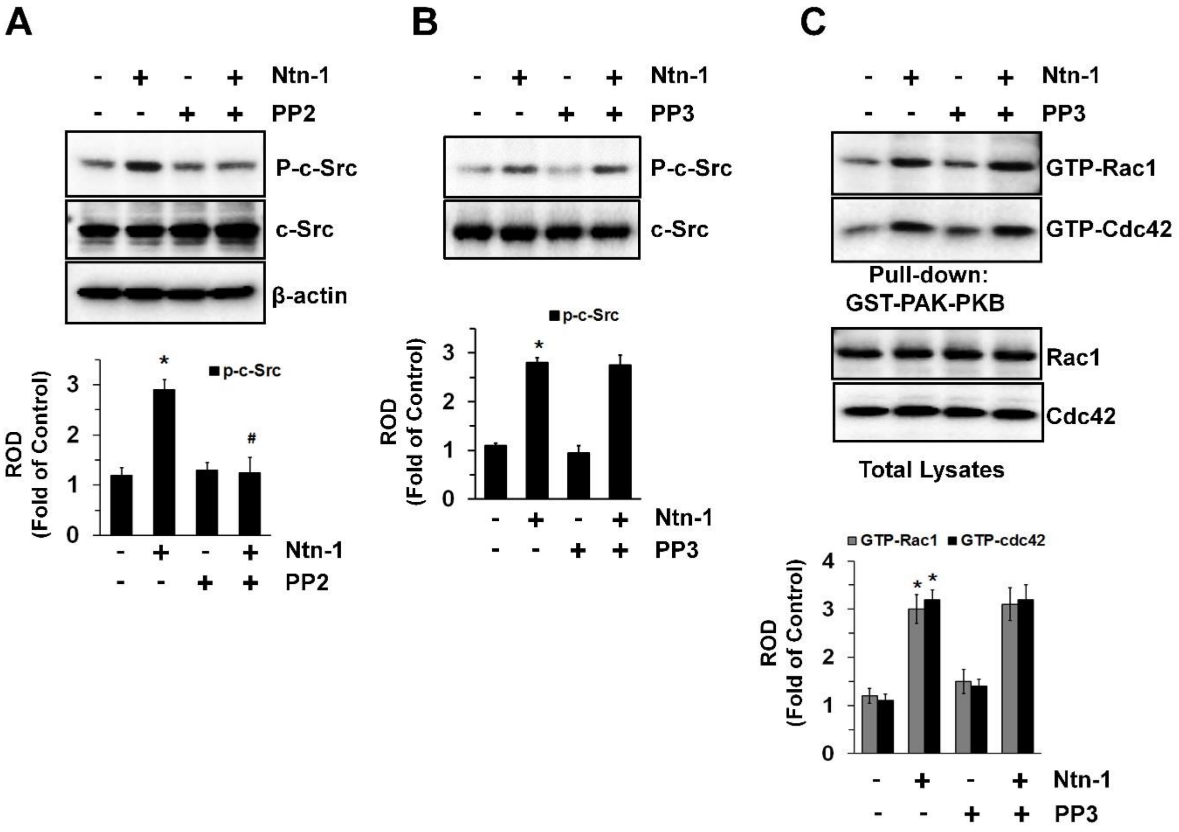

Supplementary Figure 1. Effect of PP2 and PP3 in c-Src, Rac1, and Cdc42 activation. Phosphorylation of c-Src in cells pre-treated with either PP2 (10 μM) (A) or PP3 (10 μM) (B) for 30 min prior to Ntn-1 exposure for 30 min is shown. Data represent the mean ± S.E. $n = 4$. $^*P < 0.01$ vs. vehicle. $^\#P < 0.01$ vs. Ntn-1 alone. (C) The activation of Rac1 and Cdc42 in cells pre-treated with PP3 (10 μM) for 30 min prior to Ntn-1 exposure for 30 min is shown. Data represent the mean ± S.E. $n = 4$. $^*P < 0.01$ vs. vehicle. (A-B) ROD is the abbreviation for relative optical density.

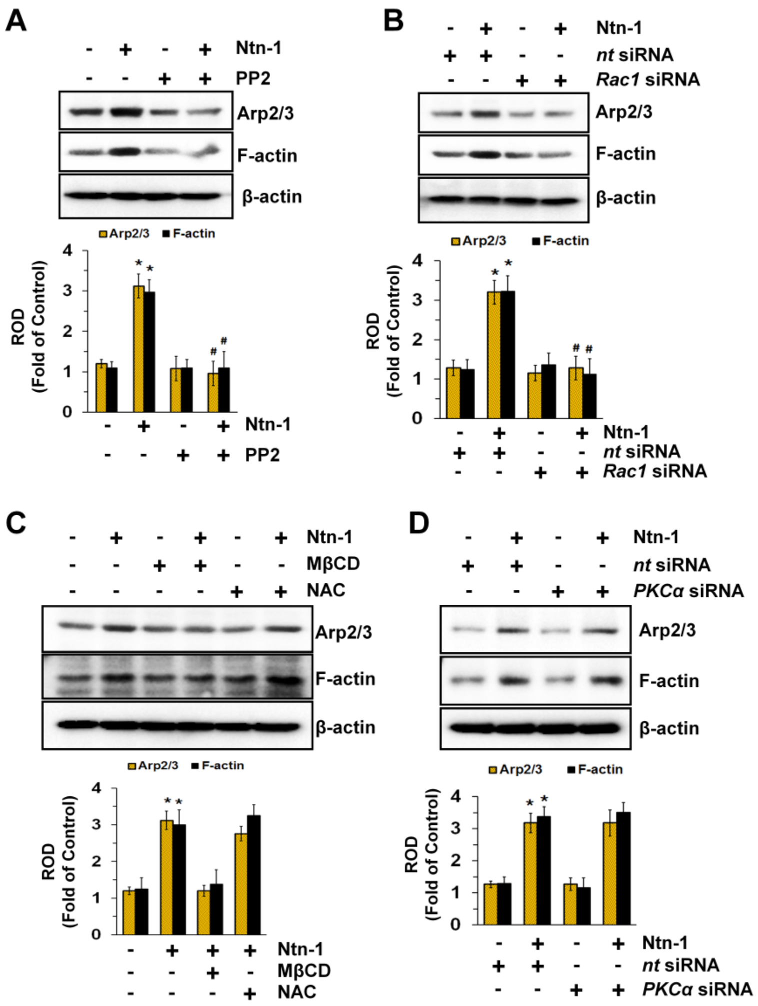

Supplementary Figure 2. Ntn-1 regulates F-actin reorganization via c-Src-mediated Rac1 activation.

(A) Cells were pre-treated with PP2 (10 μM) for 30 min prior to Ntn-1 exposure for 24 h. The effect of Ntn-1 on the expression of Arp2/3 and F-actin in cells pre-treated with PP2 is shown. Data represent the mean ± S.E. n = 4. *P < 0.01 vs. vehicle. #P < 0.01 vs. Ntn-1 alone. (B) The level of Arp2/3 and F-actin in cells transfected with *Rac1*siRNA prior to Ntn-1 exposure for 24 h is shown. Data represent the mean ± S.E. n = 4. *P < 0.01 vs. *nt*siRNA. #P < 0.01 vs. *nt* siRNA+Ntn-1. (C) The level of Arp2/3 and F-actin in cells pre-treated with MβCD (0.1 mM) and NAC (10 μM) for 30 min prior to Ntn-1 exposure for 24 h is shown. Data represent the mean ± S.E. n = 3. *P < 0.01 vs. vehicle. (D) The level of Arp2/3 and F-actin in cells transfected with *PKCα*siRNA prior to Ntn-1 exposure for 24 h is shown. Data represent the mean ± S.E. n = 4. *P < 0.05 vs. *nt* siRNA alone.

.

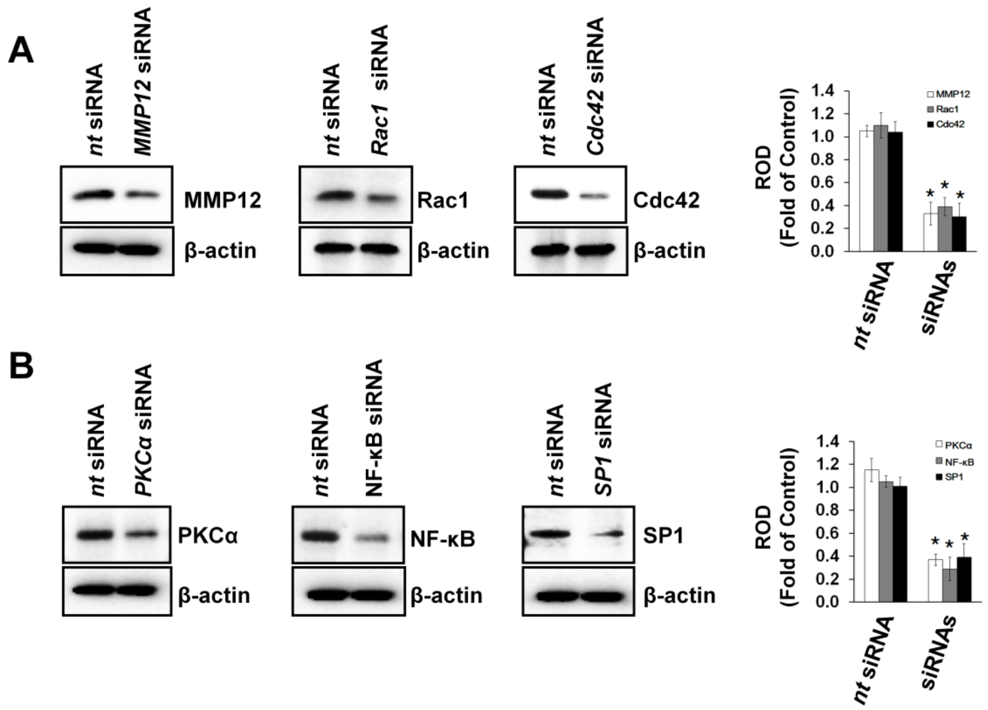

Supplementary Figure 3. Effect of siRNA on target proteins. Cells were transfected for 36 h with *MMP12, Rac1, CDC42, PKCα, NF-κBp65, and SP1*or *non-targeting* (*nt*) siRNA using HiPerFect Transfection Reagent. Protein expressions were analyzed by using Western blot. The siRNA efficacies for MMP12, Rac1, CDC42, PKCα, NF-κBp65, and SP1 determined by Western blot were 69, 65, 71, 68, 72 and 61%, respectively. Error bars represent the means ± S.E. from three independent experiments. *, $P < 0.05$ vs *nt* siRNA.
